# The Experience Sampling Method in Monitoring Social Interactions Among Children and Adolescents in School: A Systematic Literature Review

**DOI:** 10.3389/fpsyg.2022.844698

**Published:** 2022-04-04

**Authors:** Martina E. Mölsä, Mikael Lax, Johan Korhonen, Thomas P. Gumpel, Patrik Söderberg

**Affiliations:** ^1^Department of Developmental Psychology, Faculty of Education and Welfare Studies, Åbo Akademi University, Vaasa, Finland; ^2^School of Education, The Hebrew University of Jerusalem, Jerusalem, Israel

**Keywords:** experience sampling, ecological momentary assessment, intensive longitudinal data, social interaction, children, adolescents, school, systematic review

## Abstract

**Background:**

The experience sampling method (ESM) is an increasingly popular data collection method to assess interpersonal dynamics in everyday life and emotions contextualized in real-world settings. As primary advantages of ESM sampling strategies include minimization of memory biases, maximization of ecological validity, and hypothesis testing at the between- and within-person levels, ESM is suggested to be appropriate for studying the daily lives of educational actors. However, ESM appears to be underutilized in education research. We, thus, aimed to systematically evaluate the methodological characteristics and quality of published ESM studies of social interactions among children and adolescents in school settings, as well as to explore how much variance in social interaction variables could be attributed to the within-person level.

**Method:**

Using Academic Search Complete, APA PsycINFO, APA PsycArticles, ProQuest, Web of Science, Wiley Online Library, and SAGE Journals, and in accordance with PRISMA guidelines and pre-defined eligibility criteria, we conducted a systematic literature search of experience sampling studies up to November 2020. To assess methodological quality, we used a modified checklist for reporting of ESM studies.

**Results:**

Of the originally 2 413 identified studies, a final 52 experience sampling studies were included in the present review. Findings on sample and study design characteristics generally revealed wide variability. Even if high-quality studies were associated with higher scores on the training of participants in using the ESM procedure, and use of incentives, these design strategies did not reveal a statistically significant impact on compliance. The intraclass correlation coefficient was reported in nine studies and on average 58% of the variance in social interaction variables could be attributed to within-person fluctuation between timepoints.

**Conclusion:**

The current study is the first to systematically review ESM-based studies on social interactions among children and adolescents in the school context. These observations suggest that ESM is a potentially favorable technique for extracting complex social phenomena in real-world settings. We hope that this review will contribute to improving the quality assessment of ESM studies as well as to inform and guide future experience sampling studies, particularly regarding social phenomena with children and adolescents in educational settings.

## Introduction

For children and adolescents, school is a key developmental context not only for learning but also for social and emotional wellbeing (Ellis and Zarbatany, 2017). Earlier research on associations among interpersonal functioning and emotions has largely relied on retrospective studies such as large-scale questionnaires, wherein the participants are asked to report on their behavior over an extended period of time, such as the past six months. However, retrospective methodologies, where participants are required to describe his or her average or “typical” behavior, have been noted to be limited in their ability to reveal context-sensitive information (Smyth and Stone, 2003) and to be prone to memory distortion and bias (Beckett et al., 2001). This applies in particular to social experiences and interactions, whereby adolescents may switch between different participant roles both over time and within the same setting (Gumpel et al., 2014).

The experience sampling method (ESM), an intensive longitudinal data (ILD) collection technique, offers key methodological advantages to education researchers by assessing individuals in their natural environments, in real time (or close to it), and at multiple time points (Conner et al., 2009; Zirkel et al., 2015; see also Csikszentmihalyi and Larson, 1987). Compared to traditional retrospective methods, repeated sampling strategies have been argued to improve the accuracy and ecological validity by allowing for event heterogeneity, reducing memory biases, recall intervention and burden on users (Shiffman et al., 2008; Dunton et al., 2015a). Related terms for this type of assessment methodology include ecological momentary assessment (EMA; Stone and Shiffman, 1994), ambulatory assessment (AA; Trull and Ebner-Priemer, 2013), and daily diary (DD; Bolger et al., 2003). Furthermore, data may be collected by time-, signal- or event-contingent schedules, i.e., automatically at predetermined time points, automatically at random or semi-random time-points, or user-initiated after specific events (Stone and Shiffman, 1994; Shiffman et al., 2008). In the present review, the term ESM will be used throughout as an umbrella term for the family of momentary assessment techniques.

From a data analytic perspective, ESM studies allow for hypothesis testing at both within- and between-person levels (Zirkel et al., 2015; Bernstein et al., 2018; Liu et al., 2019), where the within-person approach may illuminate nuances of social interactions not typically found in traditional survey research (Bernstein et al., 2018). Analyzing data at multiple levels also avoids the ecological fallacy presented by Robinson (1950), i.e., that a correlation at one level of data does not inform us about the correlation between the same variables at another level. Multiple observations in ESM data are typically hierarchically nested within persons on either two or three levels (e.g., Scott et al., 2020). So, in a school context, participant self-reports can be nested within persons, persons within classrooms, and classrooms within schools (Zirkel et al., 2015). Furthermore, ESM designs may be employed to explore cross-level interactions, that is, the extent to which specific groups of participants are more susceptible to time-varying events than other groups, so for example whether boys' self-esteem recover faster from peer rejection than girls.

In the last decade, momentary assessment studies have become increasingly popular, not least due to the widespread adoption of smartphones and other wearables, which provide convenient and cost-effective data collection, as well as the advances in statistical software and computational processing power (van Berkel et al., 2017). Recently, a number of reviews on ESM studies have been published across disciplines related to mental health research, such as within-person assessment of social comparison (Arigo et al., 2020), quantitative and qualitative aspects of social interactions predicting within-person variance in affect (Liu et al., 2019), mood and anxiety disorders (Hall et al., 2021), and health-related behavior and psychological constructs (Williams et al., 2021). Similarly, there are best practice guidelines for conducting ESM studies (e.g., Stone and Shiffman, 2002; Trull and Ebner-Priemer, 2020; Kirtley et al., 2021). In addition, in the context of diet and physical activity among youth, Liao et al. (2016) provided a checklist for the reporting of ESM studies.

Based on these previous reviews, a gap in ESM research on social factors/phenomena in school contexts is acknowledged. Some notable reviews have illuminated within-person processes on social experiences or interactions among adults (Liu et al., 2019; Arigo et al., 2020; Mote and Fulford, 2020). Key findings in these reviews include considerable heterogeneity in assessment of social comparison processes within persons (Arigo et al., 2020), quantitative and qualitative aspects of social interactions predicting within-person variance in affect (Liu et al., 2019), and the potential of ESM in assessment of more nuanced aspects of social experiences in individuals with schizophrenia (Mote and Fulford, 2020). Another study explored the potential of ESM in education research (Zirkel et al., 2015), however from a broader perspective rather than an exclusive focus on social phenomena. According to Zirkel et al. (2015), ESM is underutilized in education research. Indeed, ESM is suggested to offer education researchers a favorable technique to investigate (complex) social phenomena within the daily lives of educational actors (the students, the parents, the staff)–areas of experiences that otherwise would be unavailable to research (Zirkel et al., 2015). Consequently, by means of ESM, no study to date has systematically reviewed momentary experiences of social interactions in samples of children and adolescents in the school context.

### Aim of Research and Research Questions

The aim of the present review is to systematically explore the state-of-the-art of ESM studies conducted on social interactions among children and adolescents in educational contexts by investigating their methodological characteristics. Since ESM studies allow for hypothesis testing at both between- and within-person levels, a secondary aim is to provide an overview of intraindividual variability of social interactions. Based on these findings, the review provides guidance on the design of future experience sampling studies.

The research questions for this review are:

1. What are the methodological characteristics of ESM studies conducted on social interactions among children and adolescents in school contexts?

2. What are the main strengths and shortcomings of the aforementioned ESM studies according to a systematic quality assessment?

3. How much variance of the social interaction variables can be attributed to the within-person level?

## Materials and Methods

### Literature Search Strategy

The Preferred Reporting Items for Systematic reviews and Meta-Analyses (PRISMA; Moher et al., 2009) was followed during the search for and selection of studies. To perform the search, we used the electronic databases Academic Search Complete, APA PsycINFO, APA PsycArticles, ERIC, ProQuest, Web of Science, Wiley Online Library, and SAGE Journals. We used combinations of the following search terms and Boolean operators within the abstract of a given study: (“experience sampl^*^” OR experience-sampl^*^ OR “ecologic^*^ momentary” OR “momentary assess^*^” OR “sampling method^*^” OR “intensive longitudinal method^*^” OR “intensive longitudinal assess^*^” OR “intensive longitudinal research” OR “daily diar^*^” OR “diary method^*^” OR “diary stud^*^” OR “electronic diar^*^” OR “ambulatory assessment” OR “ambulatory monitoring” OR “ambulatory measur^*^”) AND (social^*^ OR interact^*^ OR communicat^*^ OR interpersonal) AND (student^*^ OR pupil^*^ OR peer^*^ OR classmate^*^ OR classroom^*^ OR school OR college OR education^*^ OR academic).

We used filters to limit our search results to English language articles published in peer reviewed journals. No date limits were imposed. The last search was conducted on November 16, 2020. Additional studies not found through initial search of the databases were identified by reviewing the reference lists of recent ESM reviews (e.g., Liu et al., 2019). An auto-alert service was set up in Academic Search Complete (until April, 2021), ProQuest (until January, 2021), Wiley Online Library (until July, 2021), and SAGE Journals (until June, 2021) for notification of any related articles matching the search terms.

### Eligibility Criteria

To determine the eligibility of the studies, two authors (MM and ML) reviewed the titles/abstracts, and full articles independently, while applying the following selection criteria: (1) Children and adolescents aged 5 to 17 years; (2) Social interactions (including social experiences and behaviors) were measured quantitatively by ESM (i.e., use of fixed items or quantitative coding of open-ended questions), either by momentary measures or by end of the day reports; (3) School context, pertaining to primary and secondary education (grade 1–12, according to US/International grades); and (4) Not being a review, meta-analysis, case study, protocol, dissertation, or conference abstract.

The first 100 citations were used to pilot-test the screening process iteratively until an acceptable level of inter-rater reliability (IRR) of Cohen's Kappa > 0.8 was achieved (Landis and Koch, 1977). Once the IRR level was acceptable, the screening process continued until 40% of all unique citations were coded either as “Yes”, “No” or “Maybe”. After discussing disagreements, the IRR between the two authors screening the first 40% of the abstracts was 98% (κ = 0.98). After 100% of the manuscripts were screened, any remaining disagreements, including citations with the “Maybe” code, were discussed within the research group, and by final decisions made by senior researchers (PS and JK), consensus on inclusion/exclusion was reached.

### Data Extraction and Study Quality Assessment

The extracted data included sample characteristics, data collection procedures, and study design. In addition, results from statistical analyses related to social variables assessed by ESM were recorded in an excel spreadsheet. Missing information was coded as N/A (not available). Data extraction was conducted by two authors (MM and PS) and disagreements were resolved by consensus-based discussions.

To the best of our knowledge, there is no specific tool for assessment of quality of ESM studies. For the present review, existing quality assessment instruments were considered unsuitable, since most of them are designed for evaluating clinical trials (Sterne et al., 2016; e.g., Higgins et al., 2021) or for specific study designs (e.g., Critical Appraisal Skills Programme UK, n.d.; National Heart Lung and Blood Institute., n.d.). Therefore, quality was assessed for each study by using a modified version of the Checklist for Reporting of EMA Studies (Liao et al., 2016; Mason et al., 2020; see also Degroote et al., 2020; CREMAS); derived from an adapted STROBE (Strengthening the Reporting of Observational Studies in Epidemiology; von Elm et al., 2008) checklist for reporting. Two authors (MM and ML) independently assessed methodological quality according to the following items: (1) Training of participants for EMA protocol, (2) Technology used for EMA, (3) Wave duration, (4) Monitoring period, (5) Prompting schedule, (6) Prompt frequency, (7) Latency, (8) Attrition, (9) Compliance rate, (10) Missing data, and (11) Limitations (see **Table 2**). These items, in the CREMAS, are described in detail in the Supplemental Material 1. Each study received 0 or 1 point per item, except for the item Technology that was divided into two sub-items worth half a point each. (For momentary studies, 0.5 p were given for reporting of device and/or model, and an additional 0.5 p were given for reporting of operating system and/or ESM program name. For diary studies, 1 point was given if format, i.e., paper or electronic form, was reported for.) Score disagreements were resolved through discussion and with input from a third author (PS).

Intraclass correlation coefficient (ICC) is the proportion of variance in the outcome that is between (vs. within) persons; 1–ICC gives the variance within persons. We calculated how much variance of the (extracted) social interaction variables were attributed to the within-person level accordingly.

## Results

**Figure 1:**
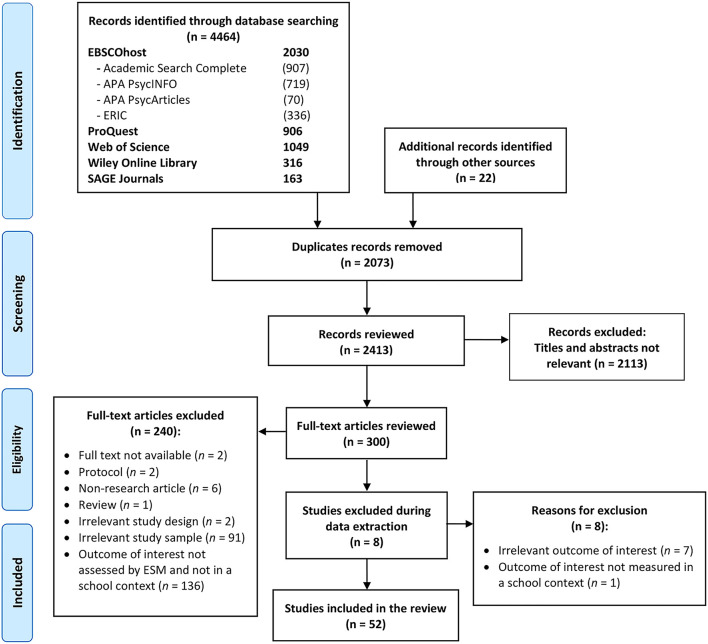
PRISMA flow diagram for systematic review. Source: Moher et al. (2009).

The literature search yielded 4,486 studies, of which 2,073 were duplicates, leaving 2413 to be screened for eligibility. A total of 52 studies fulfilled all the criteria and were included in the review (see Figure 1 for the article selection process). The earliest study was published in 1996, but the great majority of studies were published between 2002 and 2020.

Data extraction resulted in 118 main variables, including on the one hand reports on the social context (for example, “are you alone or with someone right now”; 51 variables) and on the other hand measures on individual social experiences and behaviors (for example, “have you been mean to someone within the last hour”; 67 variables). Hence, in the present study, social interactions include measures of social contextual factors, and social experiences including social behaviors.

### Sample and Design Characteristics

Sample and study design characteristics are presented in Table 1. The studies varied greatly in sample size, with a range from 10 (Vilaysack et al., 2016) to 828 (Csikszentmihalyi and Hunter, 2014) participants (*M* = 200, *SD* = 183.33; grand total = 10 317). About half (56%) of the studies included adolescents only (age 13–17), 15% included children only (age < 13), 15% included both children and adolescents, whereas 13% of the studies noted that participants were, for example, primary school students but did not report participant age. All but one study reported participant gender; and while most samples were within a 40/60 split between boys and girls, a quarter of the studies reported 60% or more girls.

**Table 1:** Characteristics of included studies (*N* = 52).

**References**	**Sample size**	**Mean age /range**	**% males**	**Terminology**	**Format**	**Sampling scheme**	**Days**	**Obs per day**	**Total obs**	**Compliance**	**Incentives**
Alarcón et al. (2020)	52	M = 16.2; R = N/A	45 %	EMA	Phone call	Time	10	2	501	86.5%	N/A
Helgeson et al. (2009)	76	M = 14.5; R = 13–16	40 %	EMA	Palm pilot + beeper	Time	4	2–9; M = 7	M = 2,128	78 %	Fixed
Rusby et al. (2013)	82	M = N/A; R = N/A; (7th graders)	38 %	EMA	Electronic device	Signal	21	3–4 per wave	Wave 1: 4,981, Wave 2&3: 4583	On average: Wave 1: 75%, Wave 2&3: 69%	Fixed + Incremental
Weinstein and Mermelstein (2007)	517	8th grade: M = 13.9; R = N/A 10th grade: M = 16; R = N/A	N/A	EMA	Palm pilot	Signal	7	5–7	N/A	85 %	Fixed
Weinstein et al. (2006)	562	M = 14.4; R = N/A	37 %	EMA	Palm pilot	Signal	7	5–7	48,892	85 %	Fixed
Dunton et al. (2007)	524	M = 14.5; R = 11–16	50 %	EMA	Palm pilot	Time	4	25–30	M = 12,733	On average: 82.5%	Incremental
Dunton et al. (2015b)	20	M = N/A; R = 12–17	44 %	EMA	Smartphone app	Signal + Event	7	N/A	655 (Signal: 462 Event: 193)	On average: 50.1%	Fixed + Incremental
Morrow et al. (2018)	182	M = 10.6; R = N/A	58 %	EMA	Paper	Time	8	1	1,422	On average: 97%	Fixed
Rivenbark et al. (2019)	395	M = N/A; R = 10–16	44 %	EMA	Smartphone app	Unclear	14	3	13,017	80 %	N/A
Slot et al. (2019)	42	M = N/A; R = 14–16	43 %	ESM	Smartphone app	Time	14	max. 8	2,642	On average: 62.9%	Incremental
van Roekel et al. (2013)	303	M = 14.2; R = 13–16	Group 1: 40%; Group 2: 45%	ESM	Smartphone app	Signal	6	9	10,865	On average: 68.5%	Incremental
Vilaysack et al. (2016)	10	M = 6.25; R = 5–7	N/A	ESM	Smartphone app	Signal	7	8	N/A	On average: 47.6%	N/A
Bassi and Delle Fave (2004)	Group 1: 60; Group 2: 60	M = N/A; R = 15–18	49 %	ESM	PDA + paper	Signal	7	6–8	4,567	N/S	N/A
Csikszentmihalyi and Hunter (2014)	828	M = N/A; R = N/A (6th, 8th, 10th, 12th graders)	52 %	ESM	Wristwatch + paper	Signal	56	8	N/A	68.1%	N/A
Ha et al. (2019)	286	M = 14.2; R = 13–16	50 %	ESM	Smartphone app	Time	Unclear (max. 6)	9	11,056	69.8%	Incremental
Henker et al. (2002)	155	M = 14.5; R = 13–16	53 %	ESM	Palm pilot	Time	4 per wave (8 in total)	25–30	26,418	On average: 80%	Incremental
Jessup et al. (2017)	12	M = N/A; R = 13–17	50 %	ESM	Smartphone app	Signal	7	7	N/A	On average: 69%	N/A
Johnson and Swendsen (2014)	35	M = 12.1; R = 12–13	50 %	ESM	Palm pilot	Time	7	4	N/A	On average: 80.2%	Incremental
Larson (1997)	483	M = N/A; R = 10–15	31 %	ESM	Beeper + paper	Signal	7	7	18,022	90 %	Fixed
Moneta and Csikszentmihalyi (1996)	208	M = N/A; R = 14–17	40 %	ESM	Beeper + paper	Signal	7	8	7811	On average: 59%	N/A
Perez (2011)	796	M = N/A; R = N/A (6th to 12th graders)	42 %	ESM	Beeper + paper	Signal	7	8	22,335	83.3%	N/A
Shernoff and Vandell (2007)	165	M = N/A; R = N/A (8th graders)	50 %	ESM	Beeper + paper	Signal	7 per wave (14 in total)	5	1596	On average: 94%	Incremental
Slot et al. (2020)	44	M = N/A; R = 14–16	37 %	ESM	Smartphone app	Unclear	14 per wave (56 in total)	Max. 8	11,059	65.5% (across waves)	Incremental
Tavares et al. (2019)	245	M = 16.6; R = 14–16	57 %	ESM	Electronic device + paper	Signal	7	8	N/A	On average: 61%	Fixed
Uink et al. (2016)	108	M = 14.7; R = 13–16	43 %	ESM	Smartphone	Signal	6	5	3240	On average: 56.7%	None were given
van Roekel et al. (2014)	303	M = 14.2; R = 13–16	43 %	ESM	Smartphone app	Signal	6	9	10,865	On average: 68.5%	Incremental
van Roekel et al. (2015)	303	M = 14.2; R = N/A	31 %	ESM	Smartphone	Signal	6	9	10,865	69 %	Incremental
Verma et al. (2002)	100	M = 13.3; R = N/A	41 %	ESM	Beeper + paper	Signal	7	8	4764	On average: 85.9%	N/A
Whalen et al. (2002)	153	M = 14.5; R = 13–16	41 %	ESM	Palm pilot	Time	4 per time point (8 in total)	25–30	T1+T2: 26,418	On average: 80%	Incremental
Zurbriggen et al. (2018)	120	M = 15.8; R = N/A	41 %	ESM	Smartphone	Signal	7	6	3930	74.6%	N/A
Rathunde and Csikszentmihalyi (2005)	Group 1: 140 Group 2: 150	M = N/A; R = N/A (6th and 8th graders)	49 %	ESM	Beeper + paper	Unclear	7 per group	Group 1: 8 Group 2: 8	N/A	On average: 93.8%	N/A
Steca et al. (2011)	**T2**: 130	M = 17.2; R = N/A	50 %	ESM	Electronic device + paper	Signal	7	8	2,463	N/S	N/A
Uekawa et al. (2007)	345	M = N/A; R = N/A (high school students)	M = 45%	ESM	Beeper + paper	Time	5	8–12; M = 10	2360	N/S	N/A
Chen et al. (2007)	206	M = 14.6; R = 14–16	44 %	AA	PDA and BP + HR	Time	2	Day 1: 29 Day 2: 12	4,897	80% (for diary + BP)	Fixed
Streb et al. (2015)	64	M = N/A; R = N/A (1st to 6th graders)	N/A	AA	Electronic device and HR + ECG	Time	N/A	N/A	N/A	94.2%	N/A
Bai et al. (2016)	47	M = 11.3; R = 10–13	42 %	DD	Electronic survey	Time	56	1	7,029	98 %	Incremental
Ducharme et al. (2002)	105	M = N/A; R = 15–16	40 %	DD	Paper	Time	7	1	735	70 %	Fixed
Dyches and Mayeux (2012)	217	5th graders: M = 10.8; R = N/A; 7^th^ graders: M = 12.9; R = N/A	Group 1: 50% Group 2: 40%	DD	Paper	Time	5	1	N/A	N/S	Incremental
Herres et al. (2018)	68	M = 11.2; R = 6–17	50 %	DD	Electronic survey	Time	8	1	N/A	On average: 53.5%	N/A
Pouwels et al. (2016)	**Phase 2**: 188	M = 16.3; R = N/A	40 %	DD	Electronic survey	Time	5	1	748	79.6%	Incremental
Tavernier et al. (2016)	77	M = 14.4; R = 11–18	41 %	DD	Actiwatch + paper	Time	3	2	N/A	N/S	None were given
Chiang et al. (2015)	316	M = 16.4; R = N/A	35 %	DD	Paper	Time	15	1	N/A	94%	Fixed
Griffin et al. (2019)	98	M = 17; R = 14–20	29 %	DD	Smartphone	Time	10	2	468	93.5%	Incremental
Jasini et al. (2018)	117	M = 15.6; R = 14–19	49 %	DD	Electronic survey	Time	7	1	984	On average: 78%	Incremental + Fixed
Sandstrom and Cillessen (2003)	118	M = 10.7; R = 10–13	55 %	DD	Paper	Time	7	1	826	73–89%	Incremental
Sherman et al. (2013, **Study 2**)	135	M = 12; R = N/A	50 %	DD	Paper	Time	30	1	N/A	85 %	Fixed
Douglass et al. (2016, **Study 2**)	79	M = 15.7; R = N/A	56 %	DD	Electronic survey	Time	21	1	M = 1,185	On average: 71.4%	Fixed
Santiago et al. (2016)	58	M = 13.3; R = N/A	34 %	DD	Paper	Time	7	1	N/A	84%−98%	Fixed + Incremental
Telzer et al. (2015)	93 (group 1 + group 2)	Wave 1: M = 14.8; R = 14–16; Wave 2: M = 15.9; R = 15–17	42 %	DD	Paper	Time	14 per wave (28 in total)	1	N/A	100 %	N/A
Yeager et al. (2016, **Study 2**)	205	M = N/A; R = N/A (9th graders)	50 %	DD	Paper	Event	5	1	N/A	N/A	N/A
Esposito et al. (2005)	41	M = 11.4; R = 10–13	55 %	DD	Interview	Time	7	1	N/A	N/A	N/A
Russell et al. (2016)	151	M = 13; R = 11–15	59 %	Hybrid	Smartphone + evening diary	Unclear	M = 38	3	N/A	On average: 92%	Incremental

*BP, Blood pressure, HR, heart rate, ECG, Electrocardiography, N/A, Not available, N/S, Not specified, PDA, Personal Digital Assistant, Incremental incentive, Based on completion rates of ESM prompts or diary entries during the monitoring period, participants were given an incremental amount of incentives*.

*Fixed incentive, Participants were given a fixed amount of incentives after completion of each ESM monitoring period*.

ESM-techniques varied across studies. About two thirds of the studies (*N* = 35) utilized momentary reports, described with terms such as ESM (*N* = 24), EMA (*N* = 9), or AA (*N* = 2), whereas one third of the studies (*N* = 16) were based on end of the day retrospective diary reports. One study (Russell et al., 2016) employed a hybrid design with both momentary reports and retrospective diary reports. Most studies (*N* = 29) used electronic devices (e.g., PDAs, smartphones) to collect data. The use of combined methods, such as an electronic device signaling to fill out paper-based questionnaires (*N* = 11) were the second most prevalent data collection type. Nine studies (including eight daily diaries) used solely paper-based surveys. Additionally, three studies (Chen et al., 2007; Streb et al., 2015; Tavernier et al., 2016) used some form of passive remote monitoring, however these assessments were used to assess another variable than the social dimension in the study (e.g., a wearable to measure blood pressure, heart rate, cortisol levels, or sleep episodes).

Data collection typically lasted seven days, with eight observations per day for momentary reports and one observation per day for daily diary reports. However, there was a large range of data collection days (2-56), as well as observations per day (1–30).

Most momentary report studies followed a signal-based sampling schedule, so that participants received notifications at random or semi-random times during the day (*N* = 19), whereas about a third used time-based sampling, where participants received notifications at predetermined periods (*N* = 12). Time-based sampling was either fixed (e.g., every 2 hours) or partly randomized (e.g., within 90-120 min blocks). One study employed both a signal- and event-based sampling schedule, and the remaining three momentary report studies had unclear sampling methods. Almost all daily diaries (14/16) followed a time-based sampling method. The diary surveys were completed in the evening/before bedtime (*N* = 7), during school hours/at the end of a school day (*N* = 3), twice a day (*N* = 1), or once a day without further specification (*N* = 5).

More than half of the studies (*N* = 32) reported providing incentives for participants who either partially or fully completed the study. These incentives can be categorized as incremental (i.e., based on completion rates of ESM prompts or diary entries during the monitoring period; *N* = 17), fixed (i.e., a fixed amount of money, or other rewards such as certificates or vouchers, after completion of a whole monitoring period, *N* = 11; monetary incentives ranged from US $8 to US $100), or a combination of fixed and incremental incentives (*N* = 4).

### Quality Assessment

Using the CREMAS, final quality scores for the studies ranged from 5 to 10 on a scale from 0 to 11, with an average score of 7.67 points (*SD* = 1.46). Based on CREMAS scores, each study was categorized as low (<6 points; 13 studies), medium (6.5–8.5 points; 25 studies), or high (>9 points; 14 studies) quality. Looking at individual dimensions of the assessment, most studies reported on data collection technology, wave duration (i.e., the number of waves for the study), monitoring period (i.e., the number of days each wave of the study lasted), prompting design (i.e., type of sampling scheme), prompt frequency, and compliance rate, whereas the lowest scores were found for (response) latency, and training (of participants in the ESM protocol). Quality assessments for each included study are listed in Table 2.

**Table 2:** Quality appraisal of included studies (*N* = 52).

**Author (year)**	**Training**	**Tech** **nology**	**Wave** **duration**	**Monito** **ring period**	**Promp** **ting** **design**	**Prompt frequency**	**Latency**	**Attrition**	**Compli** **ance** **rate**	**Missing data**	**Limita** **tions**	**Points (max. 11p)**	**Quality**
Alarcón et al. (2020)	0	1	1	1	1	1	1	1	1	0	1	9	High
Bai et al. (2016)	1	1	1	1	1	1	1	0	1	1	1	10	High
Bassi and Delle Fave (2004)	0	1	1	1	1	1	0	0	0	1	0	6	Low
Chen et al. (2007)	1	1	1	1	1	1	0	0	1	1	1	9	High
Chiang et al. (2015)	0	1	1	1	1	1	0	1	1	1	0	8	Medium
Csikszentmihalyi and Hunter (2014)	0	1	1	1	1	1	0	0	0	1	0	6	Low
Douglass et al. (2016)	0	1	1	1	1	1	0	0	1	0	0	6	Low
Ducharme et al. (2002)	0	1	1	1	1	1	0	1	1	1	1	9	High
Dunton et al. (2007)	0	1	1	1	1	1	0	0	1	1	1	8	Medium
Dunton et al. (2015b)	0	1	1	1	1	1	0	0	1	1	1	8	Medium
Dyches and Mayeux (2012)	0	1	1	1	1	1	0	1	0	1	1	8	Medium
Esposito et al. (2005)	0	1	1	1	1	1	0	0	0	0	0	5	Low
Griffin et al. (2019)	1	0.5	1	1	1	1	0	0	1	1	1	8,5	Medium
Ha et al. (2019)	0	1	1	1	1	1	0	1	1	1	0	8	Medium
Helgeson et al. (2009)	1	0.5	1	1	1	1	0	1	1	1	1	9,5	High
Henker et al. (2002)	1	1	1	1	1	1	0	0	1	0	1	8	Medium
Herres et al. (2018)	1	1	1	1	1	1	0	1	1	1	1	10	High
Jasini et al. (2018)	1	1	1	1	1	1	0	0	1	1	0	8	Medium
Jessup et al. (2017)	1	1	1	1	1	1	0	1	1	0	0	7	Medium
Johnson and Swendsen (2014)	1	0.5	1	1	1	1	0	1	1	1	0	8,5	Medium
Larson (1997)	0	1	1	1	1	1	0	1	1	1	0	8	Medium
Moneta and Csikszentmihalyi (1996)	0	1	1	1	1	1	0	0	1	1	1	8	Medium
Morrow et al. (2018)	0	1	1	1	1	1	0	1	1	1	0	8	Medium
Perez (2011)	0	0.5	1	1	1	1	0	0	1	1	0	6,5	Medium
Pouwels et al. (2016)	0	1	1	1	1	1	0	1	1	1	1	9	High
Rathunde and Csikszentmihalyi (2005)	1	1	1	1	0	1	0	0	1	0	0	6	Low
Rivenbark et al. (2019)	0	1	1	1	0	1	0	0	1	1	0	6	Low
Rusby et al. (2013)	1	0.5	1	1	1	1	0	1	1	1	1	9,5	High
Russell et al. (2016)	0	0.5	1	1	0	1	0	0	1	0	1	5,5	Low
Sandstrom and Cillessen (2003)	0	1	1	1	1	1	0	0	1	0	1	7	Medium
Santiago et al. (2016)	0	1	1	1	1	1	0	0	1	0	0	6	Low
Sherman et al. (2013)	0	1	1	1	1	1	0	1	1	1	0	8	Medium
Shernoff and Vandell (2007)	1	1	1	1	1	1	0	0	1	0	0	7	Medium
Slot et al. (2019)	1	1	1	1	1	1	0	0	1	1	1	9	High
Slot et al. (2020)	1	1	1	1	1	1	0	0	1	0	1	8	Medium
Steca et al. (2011)	0	1	1	1	1	1	0	0	0	0	0	5	Low
Streb et al. (2015)	0	1	0	0	1	1	0	1	1	1	0	6	Low
Tavares et al. (2019)	0	1	1	1	1	1	0	0	1	1	0	7	Medium
Tavernier et al. (2016)	0	1	1	1	1	1	1	1	0	1	1	9	High
Telzer et al. (2015)	0	1	1	1	1	1	0	0	1	0	0	6	Low
Uekawa et al. (2007)	0	1	1	1	1	1	0	0	0	0	0	5	Low
Uink et al. (2016)	0	0.5	1	1	1	1	0	1	1	1	0	7,5	Medium
van Roekel et al. (2013)	1	1	1	1	1	1	0	1	1	1	1	10	High
van Roekel et al. (2014)	0	0.5	1	1	1	1	0	0	1	1	1	7,5	Medium
van Roekel et al. (2015)	0	1	1	1	1	1	0	0	1	1	1	8	Medium
Verma et al. (2002)	0	1	1	1	1	1	0	0	1	1	0	7	Medium
Vilaysack et al. (2016)	1	1	1	1	1	1	1	0	1	1	1	10	High
Weinstein et al. (2006)	1	1	1	1	1	1	1	1	1	1	0	10	High
Weinstein and Mermelstein (2007)	1	1	1	1	1	1	0	0	1	1	1	9	High
Whalen et al. (2002)	1	0.5	1	1	1	1	0	0	1	1	1	8,5	Medium
Yeager et al. (2016)	0	1	1	1	1	1	0	0	0	0	0	5	Low
Zurbriggen et al. (2018)	0	0.5	1	1	1	1	1	0	1	1	0	7,5	Medium

Low and medium quality studies typically received less points in categories on training, attrition, latency, and limitations, and in the following we will take a closer look at each of these categories.

### Training

Studies scored a point if they reported on if, and by what methods, training of participants for ESM protocol was used. Several studies thoroughly described how participants received instructions on the ESM protocol prior to the data collection phase. For example, Slot et al. (2019) instructed participants in three steps by providing a 1.5-h instructional briefing a few months before the data collection where participants were encouraged to ask questions, a practice session to fill in the application, and an opportunity to take part in a pilot study as preparations for the assessment procedure. However, just over a third (*N* = 19) of studies described any form of training prior to the data collection phase. Most of the studies that reported on training were daily diaries (*N* = 12). An independent between-group ANOVA did not yield a statistically significant effect of training on compliance rates, *F*(1, 43) = 0.12, *p* = 0.73.

### Attrition and Compliance Rate

If studies indicated participant dropout rate throughout the study, such as in reporting attrition rates both by monitoring days and waves, the attrition criterion was reached. Regarding compliance rate, studies scored a point if they had reported the total answered ESM prompts across all subjects and the average number of ESM prompts answered per person. Thus, in ESM research, while attrition occurs when participants leave a study, (non-)compliance refers to the adherence to the ESM protocol. Consequently, attrition and non-compliance describe/lead to missingness (missing data).

Compliance rate during the ESM phase was reported in more than 85% of the studies (*N* = 45), whereas participant attrition was reported in only about a third of the studies (*N* = 19, ranging from 0% to 20%). The average compliance rate, in terms of proportion of prompts (*N* = 32), was 75% (*SD* = 12.5; mdn = 79), with a range from 48% to 94%. Looking at all studies that reported on compliance (*N* = 45), the average compliance rate was somewhat higher with 77.9% (*SD* = 13.4; mdn = 80), with a range from 48 to 100%. In five studies, some information about completed assessments were provided, but the average compliance or attrition rate across participants was unclear. Main reasons for participants dropping out included technical problems, an athletic practice or game, work, going to church, or withdrawal of consent during study. Several studies have proposed that attrition may be lower and compliance rates higher if the researchers provide some form of incentives to the participants (e.g., Rusby et al., 2013). To compare the effect of different types of incentives (fixed, incremental, combined, or not reported) on compliance rate, a one-way analysis of variance (ANOVA, *N* = 45) was computed. The independent between-group ANOVA revealed that there was not a statistically significant effect on compliance rate, *F*(3, 41) = 0.70, *p* = 0.56, for the different types of incentives.

### Latency

Latency refers to the amount of time from prompt signal to answering of prompt. Most daily diaries do not include prompt signals, and, for example, Tavernier et al. (2016) stated that there was no objective way for them to determine at what time the participants completed their dairies (daily, or retrospectively filling out diaries for several days simultaneously). Momentary reports, on the other hand, may well be affected by the timing of the signal (see Discussion section). Only six (11.5%) studies reported on latency.

### Limitations

Studies received a point if they reported limitations, including taking into account potential bias when using ESM protocol. For example, two studies discussed reactive bias, mentioning that the ESM protocol may lead youth to modify their behavior, or their attention to the daily assessments they receive (Russell et al., 2016; Griffin et al., 2019). Another study (van Roekel et al., 2015) commented on a potential bias in the use of technology in stating that the buzzing signal from smartphones, compared to the sound of a beep, could have led to more missed assessments among adolescents. Less than half of studies (48%) reported on study limitations.

### Within-Person Variability in Social Interactions

Only a fraction of the studies (*N* = 9) provided estimates of within-person variation or intraclass correlation coefficients (see Table 3). At the same time, all these studies indicated substantial within-person variability, with ICC measures on the within-person level ranging from 0.43 to 0.89 (*M* = 0.58; *SD* = 0.14; mdn = 0.52). In other words, on average 58% of the variance in social interaction variables was attributed to within-person fluctuation between timepoints rather than between-person differences.

**Table 3:** Proportion of variance at the within-person level of social interaction variables.

**Reference**	**Variables**	**Assessment of variable**	**Within-person variance**
Bai et al. (2016)	Peer problems	5 fixed items (yes/no)	0.61
Griffin et al. (2019)	Early-day peer support	3 items (4-point scale)	0.50
Griffin et al. (2019)	Early-day teacher support	3 items (4-point scale)	0.52
Pouwels et al. (2016)	Internalizing affect (lonely)	6 items (7-point scale)	0.43
van Roekel et al. (2013)	Positive company	2 items (scale unclear)	0.72
van Roekel et al. (2013)	Negative company	2 items (scale unclear)	0.66
van Roekel et al. (2014)	State loneliness	4 items (7-point scale)	0.63
Russell et al. (2016)	Antisocial behavior	6 fixed items (yes/no)	0.73
Slot et al. (2019)	Interests (e.g., socializing)	2 open-ended questions	0.89
Tavares et al. (2019)	Positive affect	10 items (7-point scale)	0.51
Tavares et al. (2019)	Negative affect	8 items (7-point scale)	0.46
Uink et al. (2016)	Lonely	1 item (5-point scale)	0.49
Uink et al. (2016)	Jealous	1 item (5-point scale)	0.43

*The values in the table are based on 1 - ICC which gives the variance within persons*.

About 20% of the high-quality studies, 19% of the medium-quality studies, and 9% of the poor-quality studies reported ICC scores for the social ESM variables. Even though this indicates that a larger proportion of high-quality studies include ICC scores, the difference was not significant, χ*2* (2, 52) = 0.66, *p* = 0.72.

## Discussion

As retrospective methodologies may be prone to recall biases and generate a simplified picture of phenomena, ESM, which enables hypothesis testing at both the within- and between-person level, has been presented as an innovative approach in assessments of daily experiences in the school context (e.g., Zirkel et al., 2015). However, no review to date has investigated ESM studies on social interactions among children and adolescents in the school context. Accordingly, we aimed to fill the identified knowledge gap by systematically exploring the state-of-the-art of ESM in studies conducted within the aforementioned scope by (a) exploring methodological characteristics, (b) assessing methodological quality, and (c) providing an overview of intraindividual variability of social interactions.

In the following sections, we discuss key findings as well as strengths and limitations of the current review. Furthermore, we provide recommendations for future ESM research on social interactions among children and adolescents in educational settings.

### Methodological Characteristics of ESM

Numerous design choices are required when planning an ESM study, including sampling frequency, durations of assessments, number of items, and whether to employ open- or closed-ended questions (Janssens et al., 2018; Doherty et al., 2020). This was evident in the current pool of ESM studies, which showed a large variation in study design, not least in terms of sample sizes, monitoring period, measurement points, and use of data collection method. In addition, the included studies show a large variation in data analysis and reporting. In particular, our quality assessment points to some key shortcomings that future studies should strive to address, such as the reporting of training, attrition, latency, and study limitations.

Inability to fully complete a data collection procedure has a negative impact on statistical power (Moskowitz and Young, 2006), and as in all longitudinal research, participant dropout is inevitable. Reasons for missing data through non-compliance in ESM data is most often systematic (Stone and Shiffman, 2002), as when participants regularly miss prompts due to work or school tasks. However, study compliance and missingness is also influenced by study design choices. For example, among the included studies, the one with the youngest participants (5-7 years) reported the lowest compliance rate (47.6%) (Vilaysack et al., 2016).

### Prompting Design

Decisions on sampling contingency (i.e., time-, signal- or event-contingent ESM) are affected inter alia by the characteristics of the sample (e.g., clinical or nonclinical sample, age group, and cultural context). Dunton et al. (2015b) suggest that adolescents in their study were less likely to respond to event-based prompts that occurred when they had recently completed a randomized survey prompt. Similarly, Himmelstein et al. (2019) suggest that as sampling burden is higher in event-based schemes, event-contingent ESM is recommended to be used for events that occur very rarely.

On the one hand, as the frequency of social interactions differ among individuals, event-contingent schedules are argued to be more suitable in capturing social interactions than signal-contingent schedules (Himmelstein et al., 2019). On the other hand, event-based schemes require active participants initiating the questionnaire themselves which can be seen as increasing participant burden, while also making the interpretation of missing data less clear, as missing data may be due to both participants choosing not to reply (i.e., selection bias) and to events simply not occurring.

Based on our findings, experience sampling with signal- and time-based sampling schemes are the most frequently used to study social interactions among children and adolescents in the educational context. Indeed, events that are continuous in nature, such as moods, are argued to be better suited for signal-based measurement (Himmelstein et al., 2019). However, an advantage with fixed-schedule sampling is the possibility to adapt survey questions to respondents in a time window when it is convenient for them. While it can be assumed that a high assessment frequency may increase missing data points following reporting burden for participants (e.g., Morren et al., 2009), a recent meta-analysis on ESM in compliance and retention over the continuum of severe mental disorders (Vachon et al., 2019), suggested that fixed sampling schedules predicted higher compliance rates. Indeed, either signal-based (randomized) or time-based (fixed) sampling schemes might be preferred if the phenomenon to be investigated is highly frequent or continuous (Himmelstein et al., 2019).

To illuminate, across studies in the present review, a high-quality study (Chen et al., 2007) using a high frequent ESM protocol (29 prompts over the course of a day), with a fixed time sampling schedule, had a compliance rate of 80%. Even if there is no “gold standard” for acceptable response rate in ESM studies, compliance rates of at least 80% have been recommended (e.g., Stone and Shiffman, 2002; Yang et al., 2019). However, since a central reason to use ESM is to reduce reporting bias, a fixed time schedule where prompts are predictable may decrease the ecological validity of the study and exacerbate reactivity effects (i.e., respondents may change their daily routines to match the sampling sequence, or their behaviors may change as a result of increased attention to the target behavior). None of the criteria used for study quality assessment in the present study, based on the CREMAS, acknowledged this issue (of anticipation).

### Training and Incentives

Before data collection, study briefing, including instructions on how to use devices chosen for data collection, may increase compliance and data quality, especially when performed individually than in groups (Palmier-Claus et al., 2011). However, for the 19 (36%) studies in the present review having reported on training, results of an independent between-group ANOVA test revealed that the effect of training on compliance rates was statistically non-significant.

Incentives may also be an important approach to maximize motivation and increase compliance rates among participants. Indeed, previous ESM reviews have shown that incentive strategies may increase compliance among young participants (Heron et al., 2017; Wen et al., 2017). These previous findings may be illuminated in our finding that use of incentives in high-quality studies was used to a greater extent than in low-quality studies (see Table 1). However, results of a one-way ANOVA revealed that none of the types of incentives had a statistically significant effect on compliance rates, which is a somewhat surprising finding since the general recommendation for increasing compliance (in ESM studies) is use of incentives (e.g., van Berkel et al., 2017). Conversely, monetary rewards may have a negative effect on data quality. For example, rewards/feedback may create unintended reactivity (i.e., participants changing their routines based on what was learned). Moreover, monetary rewards may be too low (not impacting motivation) or too high (e.g., the likelihood of participants convincing others to fill in questionnaires for them and share the incentive may be higher, see also a study where an incentive of $250 for participation resulted in low quality data; Stone et al., 1991).

The findings of statistically non-significant effects of training and incentives on compliance rates could indicate that they are not having that large of an impact as previously hypothesized. Potentially, participants (especially youngsters), may not diligently follow instructions (e.g., De Lillo et al., 2021), but rather intuitively follow the protocol. Another explanation regarding incentives could be that, during the data collection phase, researchers could have chosen to use incentives in samples where they (to a greater extent) had anticipated a greater non-response, resulting in effects canceling each other out. Moreover, as training was not reported in 33 (63.5%) studies, and incentives were not reported in 18 (34.6%) studies, it does not necessarily mean that no training or incentives were given in these studies, which is a finding we interpret as a need for more transparent reporting and updated guidelines in reporting of ESM research.

### Limitations

To a large extent, research quality is determined by study limitations (Resnik and Shamoo, 2017). Providing limitations supports proper interpretation and focus on key findings (Lingard, 2015; Ross and Zaidi, 2019). However, less than half of studies in the current review reported on study limitations regarding use of ESM, indicating (some) risk of bias and selective reporting. Furthermore, this finding may reflect the fact that ESM data, yielding multiple observations per person, requires careful management and analysis, which require methods that may not be commonly employed by researchers who typically deal with much smaller numbers of data points per participant over a comparable period of time. Multilevel modeling has been suggested to be the most appropriate method for analyzing intensive longitudinal data (Nezlek, 2008).

Regarding design issues that threaten ecological validity, ESM is, as are other types of studies, prone to limitations including validity and reliability of measurement instruments, and (non-)compliance in ESM protocol. Additionally, besides priming individuals to pay attention to certain states, the intrusive nature of the ESM experience has been suggested to change individuals (Napa Scollon et al., 2009). These measurement distortions are more likely when the behavior being reported has a clearly positive or negative valence, as is often the case when examining social-emotional behavior (Barta et al., 2012; Fisher and To, 2012). In the present review, two (3.8%) out of 52 studies (Russell et al., 2016; Griffin et al., 2019) explicitly commented on this external validity problem. Clearly, until issues of reactivity are better investigated, and its parameters understood, we cannot be certain that ESM is indeed tapping into an objective phenomenon, or whether it has been transformed by measurement (Napa Scollon et al., 2009).

### Latency and Other Design Features

Since the validity of ESM studies is based on momentary experiences, reporting of latency (time difference) should be principal (e.g., de Vries et al., 2021). Indeed, a smaller time lag between signal and response improves the quality of the data (e.g., Scollon et al., 2003). However, short response latencies could also be indicative of response effects (Mayerl, 2013). Whereas short response latencies may indicate disagreement with negative items, fast response latencies may indicate agreement with positive items. Short response latencies could also be a result of random responses (Mayerl, 2013). Nonetheless, 88.5% of studies in the present review did not report the latency between the beep and participant response.

Related aspects to consider include response time, i.e., how long a participant can respond to signal-activated questionnaires (e.g., 10 min or 1 h), and how much time it takes to log the questionnaire (e.g., within 1–2 min). Indeed, since the goal of ESM is to capture momentary ratings of experiences, it may be prudent to determine the maximum allowed delays as well as software capabilities (e.g., use of notification expiration) when designing/evaluating an ESM study. Access to a (reliable) internet connection may also affect protocol adherence and dropout.

Other protocol issues include types of items (e.g., use of single- or multi-item scales), and the number of questions that can be asked per assessment/prompt without compromising compliance and the quality of data retrieved from the participants. Other considerations include whether redundant items are used in the questionnaire (which may cause unnecessary participant burden). Are cross-sectional questionnaires used (and modified) for the ESM protocol, and how are the items phrased? Considerations of within- and between-person variation, as well as the disaggregation of within- and between-person effects, is required in assessment of (item) validity in ESM research (e.g., Shrout and Lane, 2012). However, as the reporting of items in the reviewed studies was both scattered and sparse, we cannot comment on validity and reliability aspects concerning use of negatively and positively worded items used in social interaction ESM surveys (i.e., the valence of wording can pose a threat to internal consistency of scales; e.g., Zeng et al., 2020). Furthermore, using more than one temporal contextualization of responses (e.g., *right now* vs. *in the last day*) may be problematic (Singh and Björling, 2019), leading to differences in reported affective states (Stone et al., 2020). Consequently, failure to address these issues puts the validity, reliability, and replicability of ESM studies at risk.

In summary, due to high methodological heterogeneity in ESM studies, and to reduce potential threats of ecological/external validity (and to promote high-quality ESM research), we, in accordance with findings in previous ESM publications (e.g., Zirkel et al., 2015; Himmelstein et al., 2019; van Roekel et al., 2019; Hall et al., 2021), emphasize the importance of a complete and transparent reporting of study design choices and results, such as in use of items, training of participants, response latency, and study limitations, not least when it comes to compliance rates and attrition. Moreover, study design characteristics such as training of participants on ESM protocol, use and type of incentives may be relevant for the association with attrition and compliance. However, our findings on the association among training, incentives, and compliance, indicated opposite effects.

### Evaluation of Quality Assessment

As there are no existing standardized quality assessment checklists used for ESM research, we chose to assess the quality of the studies following procedures (and criteria) used in a previous systematic review on ESM studies that used CREMAS (Liao et al., 2016; Mason et al., 2020; Papini et al., 2020; see also Degroote et al., 2020). However, it is important to note that the CREMAS is a guideline for reporting of ESM studies, and not a quality assessment protocol (see also Logullo et al., 2020). Having used the CREMAS for quality assessment of the studies in the present review, we also note that additional methodological aspects may be relevant to address in ESM studies (such as use of incentives, and types of items and questionnaires used).

Relatedly, one of the challenges of the present review was that the field of ESM is highly scattered with different conceptualizations and implementations of the ESM, and a diversity of ecological assessments that are sometimes contextualized and sometimes repeated. Within the studies included, we have observed that there is a somewhat conceptual confusion, or at least a lack of consistency with regards to how the terms for the family of momentary assessment techniques are used. However, as the research field matures, updated standards may well be established. In the meantime, we recommend aspiring researchers to adhere not only to the CREMAS but also to the additional dimensions discussed in this paper, when evaluating or designing the next ESM study.

### Within-Person Fluctuation in Social Interactions

Only a minority of studies (17%) reported within- or between-person variance for the social ESM measured variables included in the studies. Based on the limited data, the mean average variance for the ESM measured social variables was 0.42 (42% variance between-persons, 58% variance within-persons). Thus, over half of the overall variance was at daily, within-person level, suggesting that social interactions fluctuated within individuals across time points rather than children/adolescents differing from each other. Besides justifying a multilevel approach (Hox, 2010), this finding underscores the fact that these social variables (see Table 3) are highly fluctuating phenomena, and that ESM, taking the within-person level into consideration, can be a valuable tool for detecting such momentary fluctuations. However, since only nine out of 52 studies reported within- or between-person variance for the social ESM variables, this finding indicates a need for more consistent reporting strategies in analyses of ESM data.

### Strengths and Limitations

The present review makes several contributions to the existing ESM literature. First, by focusing on methodological aspects, our review addresses practical concerns about how to effectively apply ESM in studies with children and adolescents in educational settings. While notable reviews have assessed within-person processes on social interactions among adults (Liu et al., 2019; Arigo et al., 2020; Mote and Fulford, 2020), no systematic review to date has investigated social interactions among children and adolescents in the school context. Second, in terms of study quality our review discusses factors influencing missing data, and aspects of validity (e.g., non-compliance) and reliability (e.g., replicability). Third, our review contributes to the discussion on what criteria to use to evaluate the methodological quality of ESM studies.

At the same time, several limitations are worth mentioning. First, the great variation in study design in the 52 studies included makes it difficult to directly compare the studies. Because of this, and due to incomplete data in several of the papers, a meta-analysis was considered not appropriate.

Second, the inclusion of daily diaries in the review may be questioned as daily diaries differ from momentary assessments in several ways with regards to sampling schedule. Typically, diary studies sum up the whole day at the end of the day (where participants may backfill their responses) and may involve a similar type of retrospective bias as of traditional survey methods (e.g., Stone and Shiffman, 2002). However, as daily diaries share some of the essential features of ESM, such as intensive repeated assessments over time related to changing psychological states or environmental circumstances (Shiffman, 2009), daily diaries were included in the present review to ensure that as many relevant studies as possible are identified.

Third, due to no available standardized risk of bias guidelines for ESM studies, a risk of bias assessment for the studies included in the current review was not performed. However, as a part of the quality assessment of ESM studies, the reporting of limitations, including taking into account potential bias when using ESM methods, was assessed. As a next step, in a follow-up study exploring associations among social interaction variables and other correlates in the 52 studies reviewed in the present study, we will address various types of bias affecting validity in ESM research.

## Conclusion

This review is the first to summarize experience sampling methods and methodological characteristics in investigations of social interactions among youngsters in school. Overall, a considerate amount of key information was missing from studies included in the current review, indicating a need for more open and transparent reporting of ESM studies. A subset of the studies reported measures of between- and within-person variability (i.e., ICC scores) and all of these showed substantial within-person variability, suggesting that ESM is a valuable tool for studying social interactions at both the between- and the within-person level. Based on findings in the present review, and in accordance with previous research, we conclude ESM to be a favorable technique for future studies investigating within-person fluctuations (and its individual differences) within the daily lives of educational actors. The information in this review may be used by researchers designing future ESM studies to measure social phenomena among children and adolescents.

## Data Availability Statement

The original contributions presented in the study are included in the article/Supplementary Material, further inquiries can be directed to the corresponding author.

## Author Contributions

PS and TG conceived the original idea. MM performed the literature searches. MM and ML performed the screening of articles and quality appraisal of the included manuscripts. PS aided in verifying the results of the quality appraisal. MM and PS extracted data from the included manuscripts. PS and JK were involved in supervising the work. MM wrote the manuscript with input from PS, JK, TG, and ML. During the execution of the study, all authors were involved in discussions of data analysis and results. All authors contributed to the article and approved the submitted version.

## Funding

This research work was funded by the foundation Högskolestiftelsen i Österbotten.

## Conflict of Interest

The authors declare that the research was conducted in the absence of any commercial or financial relationships that could be construed as a potential conflict of interest.

## Publisher's Note

All claims expressed in this article are solely those of the authors and do not necessarily represent those of their affiliated organizations, or those of the publisher, the editors and the reviewers. Any product that may be evaluated in this article, or claim that may be made by its manufacturer, is not guaranteed or endorsed by the publisher.
